# Management outcome of thyroglossal cyst in a tertiary health center in Southwest Nigeria

**DOI:** 10.11604/pamj.2019.34.154.18765

**Published:** 2019-11-20

**Authors:** Segun Ayodeji Ogunkeyede, Olakayode Olaolu Ogundoyin

**Affiliations:** 1 Department of Otorhinolaryngology, College of Medicine, University of Ibadan, Ibadan, Nigeria; 2 University College Hospital, Ibadan, Nigeria; 3 Department of Surgery, College of Medicine, University of Ibadan, Ibadan, Nigeria

**Keywords:** Hyoid bone, neck mass, Sistrunk’s procedure, thyroglossal duct cyst

## Abstract

Thyroglossal duct cyst is a non-odontogenic congenital developmental cyst. It is predominantly a midline anterior neck swelling in children and total excision of the tracts prevents recurrence. Retrospective hospital record analysis of patients managed with histopathology results of thyroglossal cyst between 2003 and 2018. Comparing outcomes and technique of thyroglossal cyst excision in a resource challenged environment. A total of 37 patients comprising 22(59.5%) males and 15(40.5%) females (M:F 1.4:1) with age range of 13 days to 55 years (median 6 years) were managed. The majority were children less than 10 years of age. They all presented with a fluctuant midline progressive anterior neck swelling, in addition to anterior neck ulcer 1(2.7%), discharging sinuses 3(8.1%) and thyroglossal cyst duct infections, which were managed successfully with antibiotics. Central compartment neck dissection with excision of mid-portion of the hyoid bone was performed in all the patients. Rupture of thyroglossal duct cysts was observed in 7(18.9%) at surgery, but there was no recurrence. Surgical drain was not used and most patients were discharged within 48 hours postoperatively. Thyroglossal duct cyst was confirmed at histology without any evidence of mitotic changes. There was no recurrence for the Sistrunk's procedure in all specialties. The modification of the Sistrunk's procedure with mid-anterior neck dissection is effective in excising a thyroglossal duct cyst, hence preventing recurrence. Non-usage of wound drains and short hospital stay are cost effective.

## Introduction

Thyroglossal duct cyst (TGDC) is a congenital problem due to the inability of thyroglossal duct to disappear during intrauterine development [1]. The thyroid gland develops from the median outgrowth of primitive pharynx at third week of gestation and it descends via the thyroglossal tract to the neck, passing in the front of the body of hyoid bone. It reaches its final position in the anterior neck inferior to the thyroid cartilage in the inferior pre-tracheal neck by the seventh week of gestation [2, 3] . The thyroglossal tract usually disappears by the 10^th^ week of gestation [4]. However, approximately 7% of the general population have persistent duct and cystic degeneration of the duct form TGDC [3, 5]. TGDC typically occurs before the age of 10 years but may occur in substantial minority of young adults [6, 7] where they often appear after respiratory tract infection. It can occur along the route of migration of the thyroid gland from foramen cecum to the anterior neck [5]. It presents clinically as painless, slow progressive mobile midline anterior neck swelling, which moves vertically with swallowing or protrusion of tongue, due to their attachment to the hyoid bone [1]. TGDC can exist as cysts in the thyroid gland, laryngeal frame work, hyoid bone, lateral side of the neck and the tongue, thus presenting with respiratory and vocal symptoms [8]. Rarely, it may be secondarily infected, or associated with a fistula [1]. Thyroglossal duct cysts are mostly infra-hyoid in location, though it can be at hyoid or suprahyoid level [9]. Sistrunk's operation is the definitive treatment, this involves the excision of the TGDC with its tract extending to the foramen caecum along with the mid-portion of hyoid with which the tract is intimately related to ensure total excision of the remnants of thyroglossal duct [10]. This is to avoid incomplete excision that may result in recurrence [11]. This study presents the characteristics of thyroglossal duct cyst in children and the management outcome in our Centre.

## Methods

This is a retrospective review of clinical records and histological reports of all patients diagnosed with thyroglossal duct cyst in the Departments of Otorhinolaryngology and Surgery, University College Hospital, Ibadan, Nigeria, over a period of 15 years (January 2003-January 2018). The institution is a tertiary health institution and manages patients referred from primary, secondary and other tertiary health institutions in Nigeria. The hospital surgical registers, patients' case notes and operation notes were retrieved. The data extracted with a structured questionnaire included the patients' demography, clinical presentation, presence or absence of preoperative infection, diagnostic methods, imaging modality, location of the lesion, surgical management, postoperative complications and recurrence rate. All cases were diagnosed by using the clinical features of the disease. Socioeconomic status of the patients was based on parental occupational strata [12]. Data collected were entered into the statistical package for social sciences computer software (IBM-SPSS Version 20.0) for descriptive analysis and results are presented in tables and figures. Level of significance was considered at p < 0.05 at 95% Confidence Interval.

## Results

A total of 37 patients comprising 22(59.5%) males and 15(40.5%) females (M:F 1.4:1) were managed during this period. Their ages ranged from 13 days to 55 years (median 6 years). Majority of the patients were less than 10 years of age and from low socioeconomic status 26(70.2%). They all presented with a fluctuant midline progressive anterior neck swelling. One (2.7%) patient had an associated anterior neck ulcer whereas 3(8.1%) had discharging sinuses and they were all children (Table 1). The duration of the symptoms prior to presentation varied from 1 week to 6 years (median: 6months). All the patients were referred by the family physicians; of which 3(8.1%) cases had incision and drainage of TGDC, but were referred due to recurrence of the cyst. Seven (18.9%) patients had TGDC infection prior to presentation in the hospital. Of these, infected sinus was observed in 2(5.4%) patients and infected cysts in 5(13.5%) patients. Four (10.8%) of these patients were managed successfully with antibiotics by the family physicians and 3(8.1%) were referred. They were, however managed with antibiotics for 5-11 days before surgical excision.

**Table 1:** Socio-demographic data of the patients

Factors		Frequency (%)
**Age (years)**	≤ 10	26(70.3)
	11-20	8(21.6)
	21-30	2(5.4)
	31-40	0(0)
	41-50	0(0)
	51-60	1(2.7)
**Sex**	Male	22(59.5)
	Female	15(40.5)
**Socioeconomic status**	High	0(0)
	Middle	3(8.1)
**Site of cyst**	Low	34(91.9)
	Supra-hyoid	9(24.3)
	Hyoid	0(0)
	Infra-hyoid	28(75.7)
**Other associated factors**	Sinus	3(8.1)
	Ulcer	1(2.7)

TGDC were supra-hyoid in location in 9(24.3%) patients and infra-hyoid in 28(75.7%) patients. Ultrasound reports showed cystic mass and normal thyroid gland in all the patients, except 5(13.5%) cases that had no ultrasound report. The interval between hospital presentation and surgical excision of TGDC varies from 3 weeks to 9 months (mean 2-months). Nine (24.3%) patients were managed by otorhinolaryngologists and 28(75.7%) by paediatric surgeons. None of the patients were co-managed by the two specialties. The primary surgeons were consultants in 13(35.1%) operations and registrars in 24(64.9%) cases. All the patients had cystic mass at surgery which is distinct from the thyroid gland and 7(18.9%) thyroglossal duct cysts ruptured during surgical removal. The thyroid gland was in a normal location in all patients. There was complete excision of TGDC with no attempt to identify the tract, but the mid/central portion of the hyoid bone was resected with central compartment neck dissection to the glosso-hyhoid muscles. Surgical drain was not used in any of the cases and none had postoperative complications or recurrences during the follow-up, although, 8(21.6%) patients did not come for follow-up. The average length of hospital stay postoperatively varied from 6 hours in 32(86.5%) patients to 24 hours in 3(8.1%) patients, except 2(5.4%) patients who stayed up to 5 days due to other medical conditions. The histological reports confirmed TGDC in all the patients.

## Discussion

The remnants of thyroglossal duct account for majority of anterior neck swellings in children unlike in adults [13], as observed in this study in which 70.3% were children. Although TGDC can be present at any age, it is often detected at the second decade of life [14] but majority of our patients were diagnosed in the first decade of life. This is similar to an earlier report in which the highest incidence was in the first decade of life [15]. TGDC have a bimodal age distribution with peaks at first and fifth decades of life suggesting that it is also common in the adult population [12, 16, 17] and the observed median age at presentation of 6 years is lower than the previously reported median ages of 17 and 31 years [16, 18]. This may be due to the difference in age group of the cohorts of patients in each study, with children predominating the cohort of patients in this study. This median age at presentation suggests that majority of the patients presented late in the hospital after the onset of neck swelling. This may be due to initial presentation to the general practitioners who delayed in referring them to the specialists; it may also be due to the slow progression of the mass or the use of alternative medical therapy. The delayed referral of all the patients by the general practitioners and inappropriate surgery they offered the patients suggest that there is a need for continuous medical education for general practitioners to update their knowledge on the nature of the swelling and current surgical approach. The gender difference varies from one study to another and with age. We observed male preponderance in contrast to female preponderance observed by Rupali *et al.* [17]. Whereas Thompson *et al.* observed that it predominates in male children and female adults [16]. Ren *et al.* [19] however reported no gender difference in children and adults.

Infrahyoid location of the cyst is the most commonly reported location of the cyst [14, 16, 20] with similar location in 75.7% of our patients, there was no lingual, juxtahyoid or suprasternal cysts in this study. Presentation is usually painless although pain, dysphagia and dysphonia have been reported in adults [20]. Rupture of infected cysts with formation of ulcers and sinus/fistula are less common with 8.1% of our patients presenting with fistulas [16, 17]. The ulceration and sinus discharge in our patients may be due to the use of traditional local concoction resulting in skin ulceration or the delay in hospital presentation. Other atypical presentations in previous studies include thyroglossal duct cyst with intralaryngeal extension, intralingual cyst, ruptured cyst with subsequent chronic inflammatory changes within the anterior neck compartment, thyroglossal duct cyst with intracystic solid mass, inferiorly located cyst that is mobile with deglutition but not with tongue protrusion, and thyroglossal duct cyst presenting as lateral neck swelling or airway obstruction [14, 21, 22]. The use of preoperative antibiotics targeted at oropharyngeal flora in treating infected thyroglossal duct cyst or sinus infection is the standard practice that is preferred to needle aspiration alone [23, 24] . The common bacteria isolates are staphylococcus epidermis, haemophilus influenza, and staphylococcus aureus [25]. A needle aspiration facilitates identification of the micro-organisms, decreases the size of the cyst and ease cytological analysis to rule out underlying thyroglossal duct cysts carcinoma.

Cytological evaluation with fine needle aspiration cytology (FNAC) can diagnose malignancy preoperatively, as the majority of thyroglossal duct cyst cancers are either papillary carcinoma of thyroid origin, or squamous carcinoma [1, 16]. It is impossible to identify the thyroglossal duct cyst harboring mitotic lesion, but it should be suspected if the thyroglossal duct cyst is hard, irregular and rapidly growing with palpable cervical lymph nodes [26]. None of our patients had FNAC as part of preoperative assessment before surgery because there were no clinical and radiological evidences of malignancy. FNAC determines the patients with carcinoma prior to surgery and influences decision making in offering the best care to the patients. Thus, total thyroidectomy for comprehensive loco-regional control will be performed in addition to Sistrunk's operation which is the standard surgical approach for TGDC, with subsequent postoperative adjuvant therapy [3]. The postoperative histopathological reports of the excised specimens confirmed the preoperative diagnosis of TGDC, there was no malignancy or associated thyroid gland tissue within the cyst wall. Earlier report shows correlation between preoperative and postoperative diagnosis [19], which demonstrated the appropriateness of clinical and radiological diagnosis preoperatively. Histologically, TGDC are commonly lined by a combination of respiratory and squamous epithelium [16]. Though, high incidence of thyroid gland tissue within the TGDC wall and adjacent soft tissue has been reported [16].

The diagnosis of TGDC is clinical [18], radiological and laboratory tests are important in decision making. Thyroid glands were in normal anatomical location in all patients in this study, but confusion in clinical diagnosis between thyroglossal duct cyst and the ectopic thyroid gland is well documented and may result in serious complications [27, 28]. Important clinical evaluation such as thyroid function test and radioisotope scanning can distinguish between these two entities preoperatively [29]. Neck ultrasound determines if there is thyroid gland apart from the cystic neck swelling, otherwise the patient becomes hypothyroidic after surgery if the cystic mass removed is the only thyroid gland available for the patient. Although there is no consensus on the imaging modalities for the evaluation of suspected TGDC in a patient [30], ultrasonography is the ideal method for evaluating anomalies of the neck [14, 31, 32] . It was the only radiological evaluation prior to surgery in this study. It is readily available, cheap and does not use ionizing radiation or require intravenous contrast material. Fistulography may identify the course and the number of fistulous tracts in recurrent cases; otherwise it should be avoided, as it can be complicated by fistulisation of the cyst to the skin [33]. Computerized tomography scan is rarely needed for evaluation of a presumed thyroglossal duct cyst except in atypical presentations [23].

TGDC are most commonly of high T1 signal intensity consistent with high protein content [23], but hyperintense T2 weighted magnetic resonance imaging can outline the thyroglossal duct and this is more useful in cases with atypical presentation [14]. None of our patients had recurrence or complication, probably due to the fact that Sistrunk's procedure carries low rates of complications and recurrence irrespective of the age at presentation and symptomatology [17, 18, 34]. The recurrence rate after the Sistrunk's procedure varies in practice from 1.5% to 10% [20, 23, 35]. Lack of preoperative radiological evaluation, anatomical variations such as multiple thyroglossal ducts, rupture of TGDC during dissection and surgical procedure in the presence of acute inflammation are factors that increase postoperative complications [20]. Technical errors such as insufficient resection of the duct at base of the tongue or fragmentation of the duct during a systematic search for the thyroglossal duct may also contribute to postoperative complications.

In this study all the patients had Sistrunk's operation and central compartment neck dissection, as a modification of the original Sistrunk's procedure as an effective method for excising a TGDC. This may have contributed to absence of recurrence of the cyst. Other surgical techniques such as Schlange's technique, which involves removal of the cyst and the body of the hyoid bone, is associated with a recurrence rate of 30%, whereas simple cystectomy is associated with 100% recurrence rate [36]. All the patients that had incision and drainage by the general practitioners had recurrences because the cystic sac was not removed although aspiration of the cyst can be done to reduce the size of the cyst in severe infection as part of therapeutic cares, this is prone to recurrence. Sistrunk's operation is an open procedure with obvious neck scarring [10], but endoscopic procedure is preferred using breast approach [35] or a retro-auricular incision to avoid neck scar [37], for good cosmetic outcome. This technique is not available for surgeons in resource challenged environment like Nigeria. Its advantages compared to the anterior neck incision technique have also not been clearly established. Surgical specialty and cadre of the surgeons did not influence the management outcome in this study; though Ross *et al.* reported that surgical specialty influence the management outcome and the paediatric surgeons experienced more complication than otorhinolaryngologists [20]. There was no postoperative infection, although incidence of postoperative infection as high as 41% has been reported [18].

The routine use of preoperative antibiotics for infected cases and the need to allow inflammation to subside completely before surgery may account for this. Surgical drain was not used and none of the patients had postoperative complications such as seroma or hematoma collection. This might be related to gentle tissue handling, meticulous haemostasis at surgery and probably the absence of atypical presentation of TGDC in our patients that would have warranted extensive tissue dissection. Other reported postoperative complications include vocal cord damage, hypoglossal injury, tracheal injury, dysphagia, salivary fistula, seroma, hematoma and compressive haematoma at the base of the tongue or floor of the mouth resulting in upper airway obstruction [16, 23, 38]. Laryngo-tracheal injury is a rare devastating complication of the Sistrunk's procedure, resulting in problems with the airway, swallowing, and/or voice production. It can be caused by erroneous resection of the thyroid cartilage instead of the hyoid bone or excision of intra-laryngeal TGDC. Postoperative short hospital admission stay has been reported to allow for removal of wound drain 24-48 hours after surgery [19]. Most of our patients were discharged home on the same operative day, others were observed for 24 hours after surgery but none of them had wound drain inserted. The poor follow up clinic attendance in this study may be related to low incidence of complications with no clinical problem to warrant hospital presentation. It may also be due to ineffective communication between the attending physicians and the care givers or probably the far distance of the hospital to the patients' residence.

## Conclusion

Late presentation did not affect the management outcome of TGDC but early presentation can reduce the incidence of infected cyst with sinus/fistula formation. Therefore, primary care physicians need to update their knowledge on the anterior neck congenital anomaly in order to make prompt diagnosis and referral. Ultrasonography of the neck is a reliable and suitable imaging modality for early diagnosis in most patients with typical presentations. Postoperative outcomes are good if Sistrunk operation which is the preferred procedure is performed appropriately.

### What is known about this topic

- Thyroglossal duct is responsible for majority of anterior neck swellings in children;
- It is often detected at the second decade of life, although TGDC can present at any age.

### What this study adds

- The Sistrunk operation can be done in resource challenge environments without the use of surgical wound drain;
- Patient can be discharged home on the same day of surgery, thus reducing the cost of hospital stay and without postoperative complications.

## Competing interests

The authors declare no competing interests.
